# Systemic Sclerosis With a Normotensive Scleroderma Renal Crisis: A Diagnostic Dilemma

**DOI:** 10.7759/cureus.64167

**Published:** 2024-07-09

**Authors:** Stanley E Atencah, Raheem Robertson, Nkechi Ukoha, Osahon N Idolor, James Pippim

**Affiliations:** 1 Internal Medicine, Piedmont Athens Regional Medical Center, Athens, USA; 2 Pulmonology, Piedmont Athens Regional Medical Center, Athens, USA

**Keywords:** clinical rheumatology, interstitial lung disease, renal crisis, scleroderma, systemic sclerosis

## Abstract

Systemic sclerosis (SSc), also called scleroderma, is an auto-immune rheumatic disease that is characterized by fibrosis of the skin and internal organs and vasculopathy. Three of the severe manifestations of the disease include a scleroderma renal crisis (SRC), pulmonary arterial hypertension, and digital ulceration. Vascular manifestations like Raynaud's phenomenon are an almost universal symptom in patients with SSc and are often the earliest manifestation of the disease.

An SRC occurs in approximately 10% of all patients with scleroderma. It is characterized by malignant hypertension and progressive renal failure. However, about 10% of SRC cases present with normal blood pressure or a normotensive renal crisis.

A 65-year-old man with a history of peripheral vascular disease and newly diagnosed heart failure presented to the emergency department on account of progressive discoloration of the left big toe and intermittent confusion. Initially, he was noted to be hemodynamically stable, with bluish discoloration of his left lower extremity and left big toe, which was tender to palpation with palpable distal pulses. His left toe progressively became dusky and gangrenous, necessitating ray amputation by vascular surgery. His hospital course was further complicated by worsening acute kidney injury, requiring initiation of hemodialysis, and progressive hypoxia with the transition from room air to high-flow oxygen. As part of his workup for acute kidney injury (AKI), his antinuclear antibody (ANA) was found to be positive, with high titers, as well as elevated SCl-70 IgG. Despite the initiation of hemodialysis, and post-surgical revision, he continued to deteriorate. His family opted for comfort care measures, and he died a few days later.

Although SSc is a rare disease, it is associated with significant morbidity and has one of the highest mortality rates among connective tissue diseases. SSc can present with heterogeneous manifestations, mimicking several isolated organ-specific conditions. This makes the diagnosis challenging, especially early in the course of the disease. A high index of suspicion, especially in the setting of rapidly progressing multi-organ involvement without a clear cause, should prompt further evaluation of systemic sclerosis.

## Introduction

Systemic sclerosis is a multisystem autoimmune disorder involving connective tissues and other organs of the body. It is a rare disorder with an incidence of 1.4 per 100,000 person-years and a prevalence of 17.6 cases per 100,000, mostly in women of the Black race, and though rare, it is associated with the highest mortality of all rheumatologic disorders [1-4]. This high morbidity and mortality may be attributed to delays in diagnosis associated with the challenge of recognizing the disorder, especially in the early stages and when it presents with atypical features.

In this index case, we present a diagnostic conundrum of systemic sclerosis presenting as a rapidly progressive interstitial lung disease with normotensive scleroderma renal crisis that proved to be fatal due to the delay in recognizing the diagnosis. In the presentation of this case, we aim to highlight the importance of the holistic and interdisciplinary approach to the diagnosis and care of patients presenting with unclear clinical presentation to avoid early closure and delays in the diagnosis and patient care.

## Case presentation

A 65-year-old Caucasian male with a past medical history of hypertension, peripheral vascular disease, coronary artery disease, heart failure with reduced ejection fraction (HFrEF), and obstructive sleep apnea presented to the ED. His chief complaints included progressively worsening shortness of breath, intermittent confusion, as well as pain and bluish discoloration of the left big toe and fingertips over the past six months.

The patient had been recently hospitalized for similar complaints, where he was treated for acute decompensated heart failure and delirium. A left heart catheterization revealed non-obstructive coronary artery disease after which limited guideline-directed medical therapy was initiated due to the presence of acute kidney injury and digital ischemia. He was discharged on furosemide, metoprolol, aspirin, atorvastatin, apixaban, ranolazine, and nitroglycerin paste. His family sought further management due to incomplete resolution of the left toe discoloration. In the ED, he had stable vital signs, including blood pressure at baseline and a cold, tender left great toe with persistent bluish discoloration. Laboratory studies revealed normocytic anemia with hemoglobin of 12.8 g/dL and a white blood cell count of 23 x103/nL consistent with leukocytosis (82% neutrophils and zero bands). C-reactive protein (CRP) was 2.47 mg/dL and a creatinine level at previous hospitalization of 2.53 mg/dL. His baseline creatinine was 1.3 mg/dL. Despite empirical antibiotic vancomycin and piperacillin-tazobactam, his condition continued to deteriorate. Gangrenous changes in the left great toe necessitated amputation.

**Figure 1:**
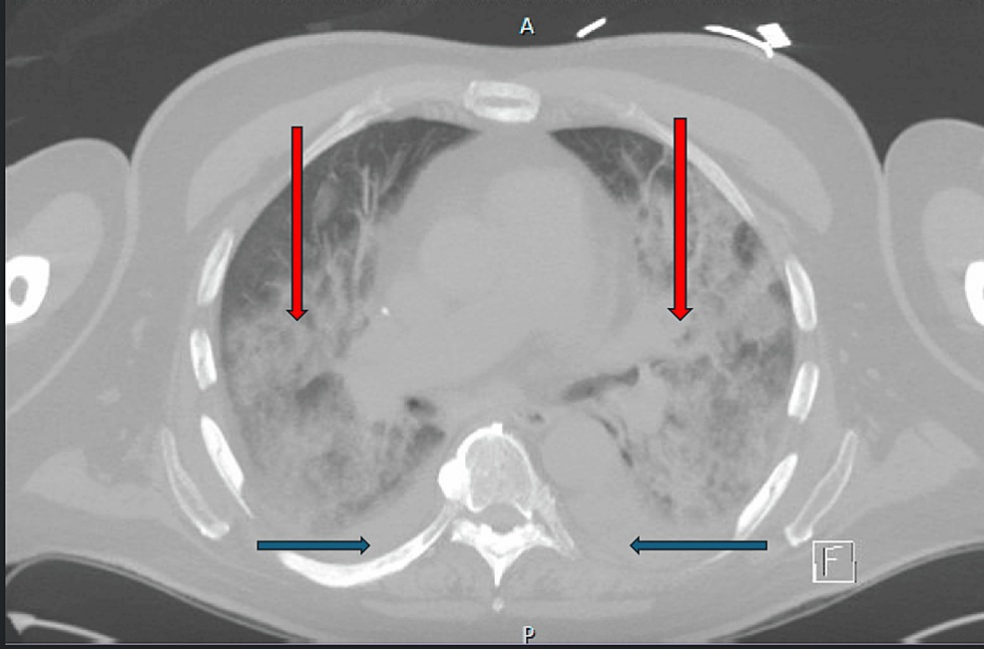
Computed tomography of the chest showing bilateral diffuse ground-glass opacities with small effusions bilaterally Blue arrows: bilateral pleural effusion; Red arrows: bilateral diffuse ground-glass opacities

Persistent acute kidney injury, possibly due to contrast-induced nephropathy and cardio-renal syndrome led to the initiation of intermittent hemodialysis, as his creatinine peaked at 3.7 mg/dl with no signs of recovery. Due to non-healing wounds, the patient had a revision amputation of the left great toe. Hypoxic respiratory failure ensued with a CT chest shown in Figure 1, revealing diffuse ground-glass opacities throughout both lungs with small bilateral pleural effusions, prompting positive airway pressure ventilation. Further serological workup shown in Table 1, revealed positive ANA with high titers 1:1280, he was subsequently started on pulse dose methylprednisone. After his SCL-70 antibody returned, methylprednisone was discontinued as steroids could be harmful with SSc. Despite these efforts, the patient's condition continued to decline with worsening respiratory failure, prompting a transition to comfort care, and he eventually died. Unfortunately, a kidney biopsy could not be performed before death due to his instability.

**Table 1:** The patient's rheumatological investigations Anti-Ds DNA: Anti-double-stranded deoxyribonucleic acid; Anti-SSA: Anti-Sjogren's syndrome-related antigen A; Anti-SSB: Anti-Sjogren's syndrome-related antigen B; Anti-Jo 1: Anti-histidyl tRNA synthetase; Anti-RNP: Anti-ribonucleoprotein; ANti-GBM: Anti-glomerular basement membrane; Anti-CCP: Anti-cyclic citrullinated peptide

Investigation	Result	Unit
Antinuclear antibody	Positive	1:1280
Anti-Ds DNA	Negative	1 IU/mL
Anti-SSA	Negative	<1
Anti-SSB	Negative	<1
Anti-Jo 1	Negative	<1
Anti-RNP	Negative	<1
Anti-Smooth muscle	Negative	<1
Anti-Chromatin	Negative	<1
Anti-SCL-70	Positive	> 8
Anti-Rheumatoid factor	Negative	11.1 IU/mL
Anti-Centromere	Negative	< 1
Anti-RNP	Negative	< 1
Anti-Myeloperoxidase	Negative	<1
Anti-Proteinase 3	Negative	<1
Anti-GBM	Negative	< 1
Anti-CCP	Negative	< 16
Cryoglobulin	Negative	Not Applicable (N/A)

## Discussion

Systemic sclerosis (SSc) is a rare autoimmune connective tissue disease characterized by skin involvement, systemic vascular pathology, and eventual multiorgan damage, especially kidneys, lungs, and heart [5]. Incidence is estimated at 1.4 per 100,000-person years with a prevalence of 17.6 cases per 100,000. It occurs most commonly in women and Black patients [3]. Although SSc is rare, it is associated with significant morbidity and has the highest mortality among all rheumatologic diseases [4]. The diagnosis can be challenging, especially earlier on in the disease course, leading to delays in diagnosis and contributing to adverse outcomes. SSc mimics several isolated organ-specific conditions making it extremely difficult to distinguish them from those conditions. A detailed clinical assessment and a high index of suspicion help to make accurate diagnoses and help avoid adverse outcomes.

The presentation of SSc is variable, however, most patients present with fatigue and Reynaud’s phenomenon [5]. The skin is the most common organ affected manifesting as puffy fingers and toes, skin thickening, limited joint mobility, skin fibrosis, calcinosis, and digital ulcers, which occur in 25-30% of patients with SSc and were present in our patient. He experienced intermittent skin thickening and digital ulcers for years before presentation [6]. The extent of skin involvement helps classify SSc into limited cutaneous, diffuse cutaneous, and sine scleroderma. However, skin thickening without Reynaud’s phenomenon makes SSc unlikely. The kidneys are also commonly affected with the most feared complication being scleroderma renal crisis, which occurs in 5-14% of patients [7,8]. This complication is almost always seen in limited to diffuse SSc and presents as sudden onset rapidly progressive worsening kidney function and malignant hypertension with hypertensive encephalopathy, congestive heart failure, microangiopathic hemolytic anemia, and thrombocytopenia [7]. It has a mortality of 20% in six months often requiring intensive care. Despite improved renal outcomes over the years with the advent of angiotensin-converting enzyme (ACE) inhibitors, scleroderma renal cell crisis remains a significant challenge due to the absence of preventative measures and the potential for rapid patient decline even with optimal medical management [8]. Our patient had rapidly deteriorating kidney function requiring hemodialysis but the absence of malignant hypertension probably decreased our suspicion of a scleroderma renal crisis. However, there have been several descriptions of normotensive scleroderma renal crisis, which may make early detection difficult and is associated with a poorer prognosis. This may have been the case with our patient who presented with a blood pressure of 130/84 mmHg, which remained in that range throughout his admission and may have contributed to the delayed diagnosis [9,10].

Six out of 10 patients with SSc will have lung involvement manifesting as interstitial lung disease, non-specific interstitial pneumonia, and pulmonary arterial hypertension [5]. Lung complications are the leading cause of mortality in SSc patients [11,12]. Of note, males and patients with a positive Scl-70 autoantibody tend to have rapidly progressive interstitial lung disease with poor prognosis as was evident in our patient [13]. He had increasing oxygen requirements to the point where he was hypoxic just by moving in bed. The gastrointestinal system is not spared, as 90% of SSc patients have some form of GI pathology, including reflux esophagitis, dysphagia, malabsorption, small intestinal bowel overgrowth, intestinal pseudo-obstruction to anorectal dysfunction, and prolapse [14-16]. Cardiac complications may include myocarditis, arrhythmias, heart failure, and focal fibrosis [5]. Arthralgias and inflammatory arthritis (12-65%), erosive arthritis, myositis (10-15%), myalgia, tendon friction rubs, and terminal acro-osteolysis (20-25%) are common musculoskeletal manifestations [17].

Diagnosis entails clinical presentation, risk classification, and laboratory assessment of autoantibodies. The American College of Rheumatology (ACR) proposed a classification system in 2013 as demonstrated in Table 2 [18].

**Table 2:** ACR/EULAR criteria for the classification of systemic sclerosis Modified from [18] ACR: American College of Rheumatology; EULAR: European Alliance of Associations for Rheumatology; MCP: Metacarpophalangeal; PAH: Pulmonary arterial hypertension; ILD: Interstitial lung disease; SSc: Systemic sclerosis

Criteria Domain	Sub-criteria	Weight
Skin thickening	Skin thickening of the fingers of both hands extending proximal to the MCP joints	9
	Puffy fingers	2
	Whole finger, distal to the MCP joints	4
Fingertip lesions	Digital tip ulcers	2
	Pitting scars	3
Telangiectasia		2
Abnormal nailfold capillaries		2
Lung involvement	PAH and/or ILD	2
Reynaud’s phenomenon		3
SSc-related antibodies		3

Several autoantibodies have been linked to SSc as shown in Table 3 and their detection not only helps diagnose the disease but also to predict certain organ involvement [5]. ANA is seen in 95% of these patients but is less specific.

**Table 3:** Autoantibodies linked to systemic sclerosis and their clinical correlates Modified from [5] SSc: Systemic sclerosis; RNA: Ribonucleic acid; Scl: Scleroderma

Autoantibodies	Clinical correlates	SSc Cutaneous type
Anti-centromere	Pulmonary arterial hypertension	Limited SSc
Anti-RNA polymerase III	Renal crises and malignancy	Diffuse
Anti-topoisomerase I (anti-Scl 70)	Progressive interstitial lung disease	Diffuse
Anti-U3 ribonucleoprotein (anti-fibrillarin)	Pulmonary arterial hypertension and myositis	Diffuse
Anti-Pm-Scl	Myositis	Limited
Anti-Th/To	Interstitial lung disease and pulmonary arterial hypertension	Limited
Anti-U1 ribonucleoprotein (anti-ribonucleoprotein)	Mixed connective tissue disease	Limited

Our patient exhibited several of the clinical features outlined above but the chronology and certainty made the diagnosis challenging, and hidden in plain sight. A diagnosis of diffuse cutaneous SSc was strongly considered, albeit later in his presentation due to digital ulcers, recent vasculopathy, rapid deterioration of kidney and pulmonary function, and positive Scl-70 autoantibody. Other challenges to early diagnosis included inadequate history from the patient due to intermittent confusion. Availability and anchoring heuristics may also have played a role, as the patient was considered to have peripheral vascular disease and/or embolic source of digital ischemia though labs and imaging findings were not in keeping with those diagnoses.

Clinicians should be aware of systemic sclerosis presenting in the non-classic demographic populations and always have a high index of suspicion especially in the setting of multi-organ involvement and inconsistent laboratory and imaging studies. If you happen to involve multiple sub-specialists in the care of a patient with multi-system disease, stop and rethink the possibility of systemic sclerosis.

## Conclusions

Systemic sclerosis can have varied manifestations and can present as isolated organ-specific diseases. A scleroderma renal crisis is a complication of systemic sclerosis that usually presents with hypertensive crises and progressive renal failure. However, some patients may be normotensive, and these subsets of patients have been noted to have poorer prognoses as compared to those with hypertensive crises. A high index of suspicion, especially in the setting of rapidly progressing multi-organ involvement without a clear cause, should prompt further evaluation of systemic sclerosis.
